# Delayed emergence due to remimazolam extravasation

**DOI:** 10.1186/s40981-022-00584-7

**Published:** 2022-12-09

**Authors:** Satoshi Uchida, Daiki Takekawa, Kazuyoshi Hirota

**Affiliations:** Department of Anesthesiology, Hirosaki University Graduate School of Medicine, 5 Zaifu-cho, Hirosaki, 036-8562 Japan

To the editor:

Remimazolam is an ultra-short-acting benzodiazepine whose sedation is rapidly reversed by the benzodiazepine antagonist flumazenil [1]. Extravasation is one of the causes of the prolonged effect of anesthetics, but the extent may depend on the absorption and elimination of each drug. We report a case of delayed emergence from general anesthesia due to remimazolam extravasation.

A 77-year-old male patient (175.6 cm, BMI 33.4 kg/cm^2^) was scheduled for thoracic endovascular aortic repair. Hepatic function was normal (AST 19 IU/L, ALT 13 IU/L). The patient showed difficult venous access due to severe obesity, but we secured an anesthetic route on his forearm. General anesthesia was managed with remimazolam and remifentanil for 3 h 5 min. The maintenance dose of remimazolam was 0.5–0.7 mg/kg/h, and 212 mg was administered in total. Although the patient received minimally invasive surgery, we had difficulty to avoid raising the patient’s blood pressure and administered remifentanil up to 0.35 μg/kg/min. Bispectral Index (BIS) value remained in the range of 60–80 during the surgery. The patient’s right-hand dorsum to forearm was obviously swollen but we could not have noticed it until the surgery was finished. We removed the venous route in consideration of the extravasation of anesthetics. However, at 45 min after the end of administration, he had not awoken or resumed spontaneous breathing. We administered 4 mg/kg of sugammadex twice in 15 min and confirmed a 100% train-of-four ratio, but still no changes were observed. We then administered 1 mg of flumazenil, which caused the patient to awaken promptly with adequate spontaneous breathing, and performed the tracheal extubation. The patient was transferred to the intensive care unit, and neither re-sleeping nor tissue dysfunction was observed in the postoperative period.

There are reports of propofol extravasation resulting in sedation or delayed emergence [2, 3], but no remimazolam extravasation has been reported. Extravasation might be noticed by higher BIS values, but we permitted it because it was one of the characteristics of remimazolam anesthesia [4]. We did not suspect that remifentanil was the cause of delayed arousal because the patient had adequate spontaneous breathing after flumazenil administration. Remimazolam was designed to be rapidly metabolized by a variety of tissue esterase. However, the kinetic behaviors of remifentanil and remimazolam are very different, with remifentanil being metabolized primarily in skeletal muscle, while remimazolam is primarily metabolized in the liver. It has been reported that kidney and lung tissue homogenates from animals metabolize remimazolam, although blood esterase does not [5]. Remimazolam could also have infiltrated intramuscularly due to extravascular leakage, but its metabolism in muscles has not been evaluated. Therefore, there is a possibility that the different kinetics resulted in a prolonged effect only for remimazolam despite remifentanil was administered through the same intravenous line. In addition, higher BMI and older age may prolong the time to extubation [6].

We experienced a case of delayed emergence due to remimazolam extravasation. The patient resumed consciousness via flumazenil antagonism. Although remimazolam is an ultra-short-acting anesthetics, the possibility of delayed emergence should be recognized if extravasation occurs.

## Data Availability

Not applicable.
